# Supplementary figures and images for: Preoperative myocardial expression of E3 ubiquitin ligases in aortic stenosis patients undergoing valve replacement and their association to postoperative hypertrophy
Source: PLoS One. 2020 Sep 18;15(9):e0237000. doi: 10.1371/journal.pone.0237000 (PMC7500680; doi:10.1371/journal.pone.0237000)

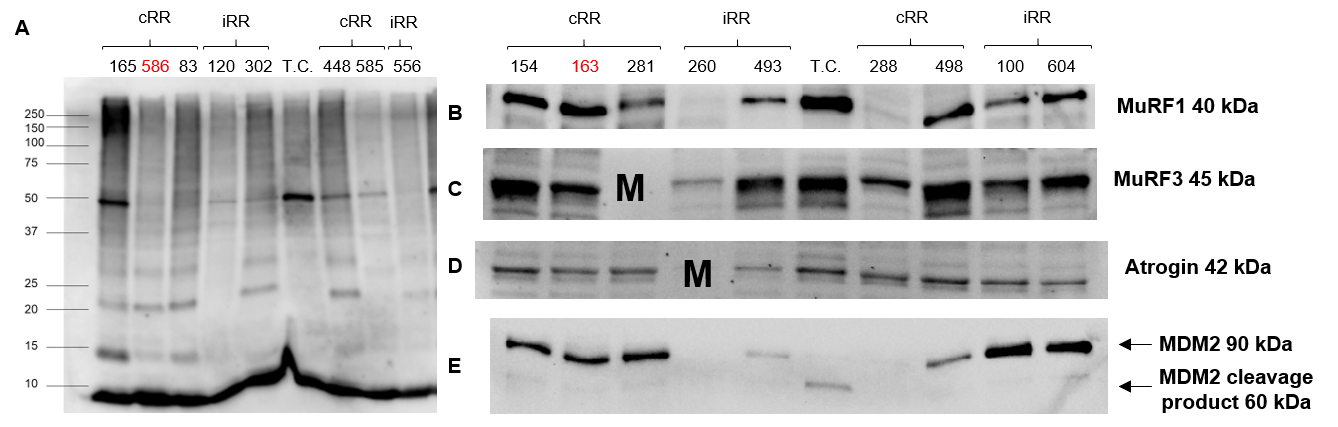

Supplement: S1 Fig — Remaining western blot scans are depicted for ubiquitin-tagged proteins (A) and for the E3 ubiquitin ligases Muscle Ring Finger (MuRF) 1 (B), MuRF3 (C), atrogin-1 (D) and Murine Double Minute 2 (MDM2) (E). The respective optical density-based semi-quantification is shown in Fig 1(F)–1(J). T.C. designates the technical control. Samples 163, 586 and 603 are marked in red because these were excluded from the study a posteriori (aortic or mitral insufficiency was found to be more or as severe as aortic stenosis). ‘M’ means missing. cRR identifies patients with complete reverse remodeling and iRR denotes patients with incomplete reverse remodeling. (TIF) [file pone.0237000.s001.tif]
