# Supplementary material for: Preoperative myocardial expression of E3 ubiquitin ligases in aortic stenosis patients undergoing valve replacement and their association to postoperative hypertrophy
Source: PLoS One. 2020 Sep 18;15(9):e0237000. doi: 10.1371/journal.pone.0237000 (PMC7500680; doi:10.1371/journal.pone.0237000)
Supplement: S1 Raw images — (PDF) [file pone.0237000.s002.pdf]

Ubiquitin western blot raw scan

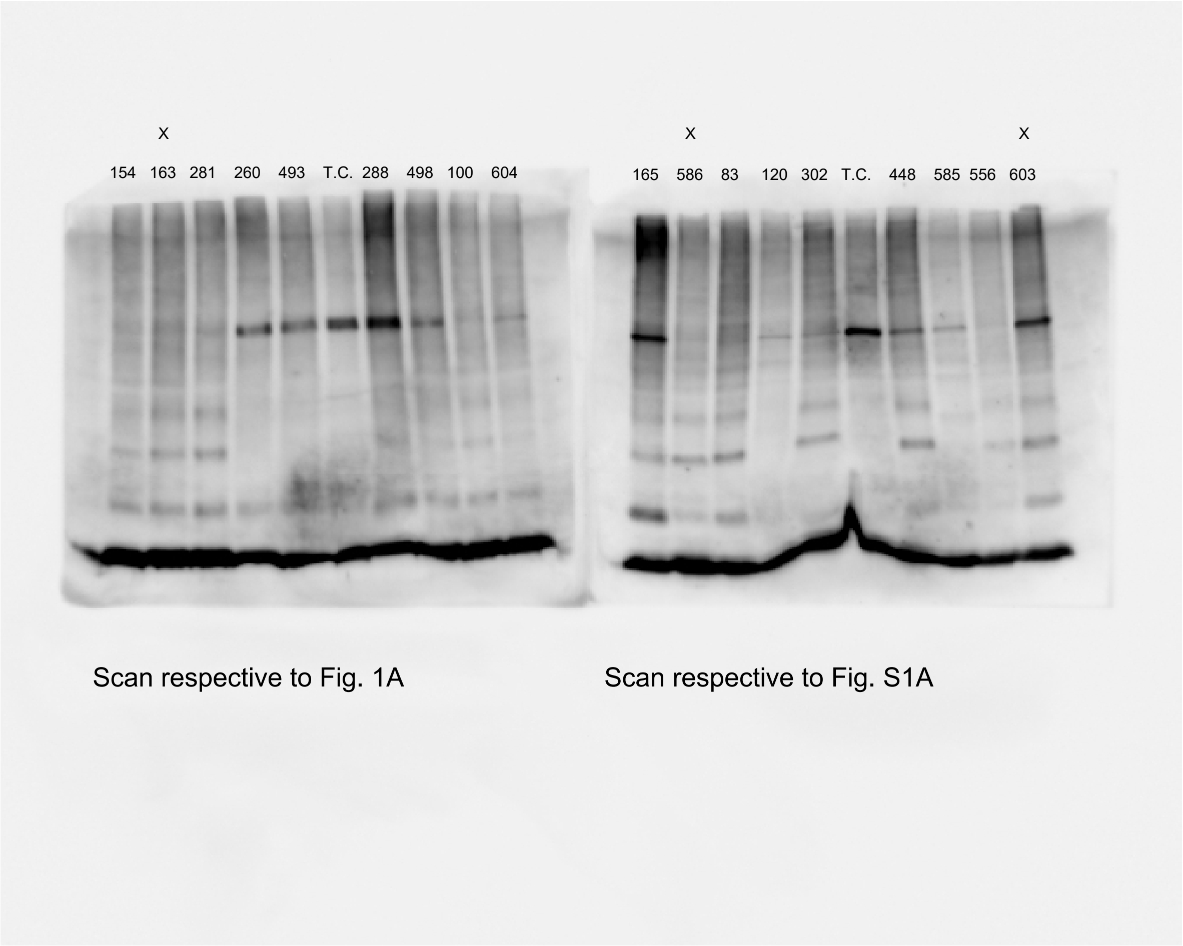

Capture method: chemiluminescence
Please see page 6, showing additionally the colorimetric signal, from which information on molecular weight ladder was collected
"x" denotes a sample not used in data analysis. However, we have not removed its lane from the Fig1A and FigS1A.

MuRF1 western blot raw scan

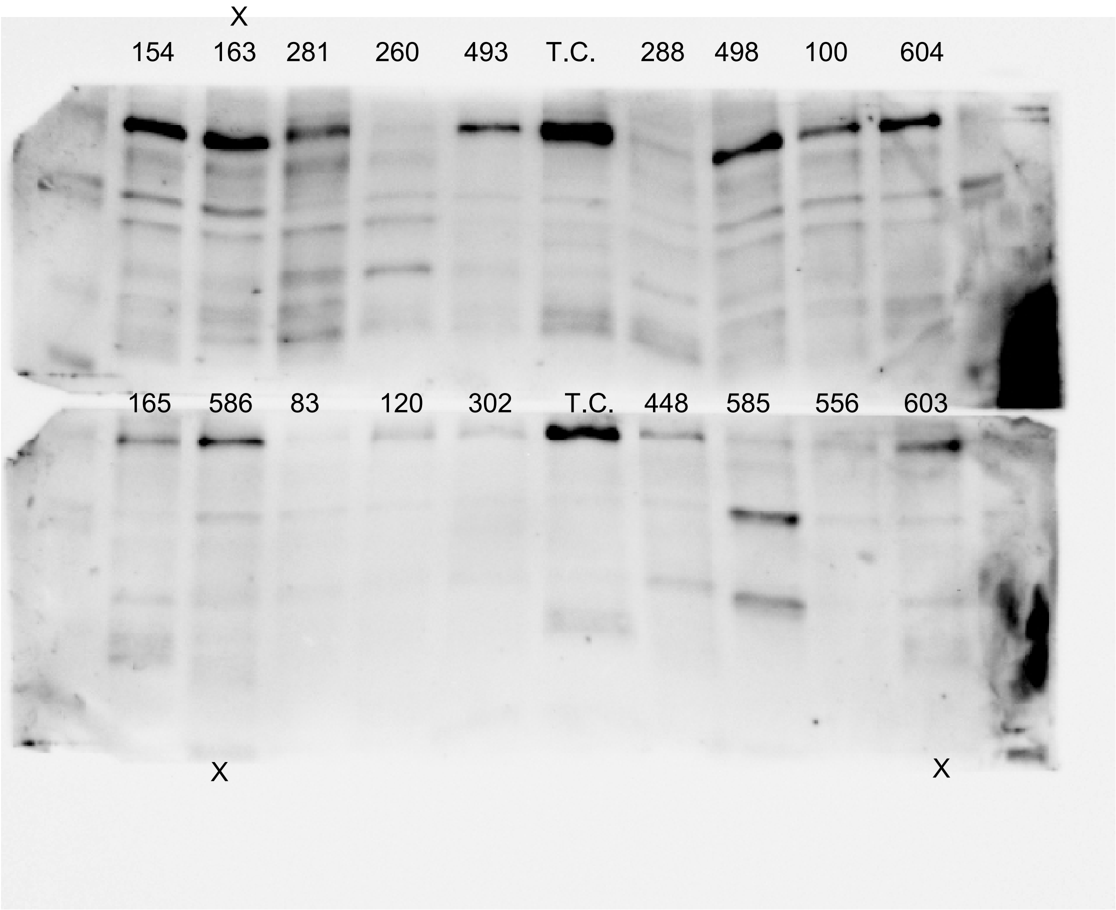

Scan respective to Fig. S1B

Scan respective to Fig. 1B

Capture method: chemiluminescence
Please see page 7, showing additionally the colorimetric signal, from which information on molecular weight ladder was collected
"x" denotes a sample not used in data analysis. However, we have not removed its lane from the Fig1B and FigS1B.
T.C. stands for technical control.

MuRF3 western blot raw scan

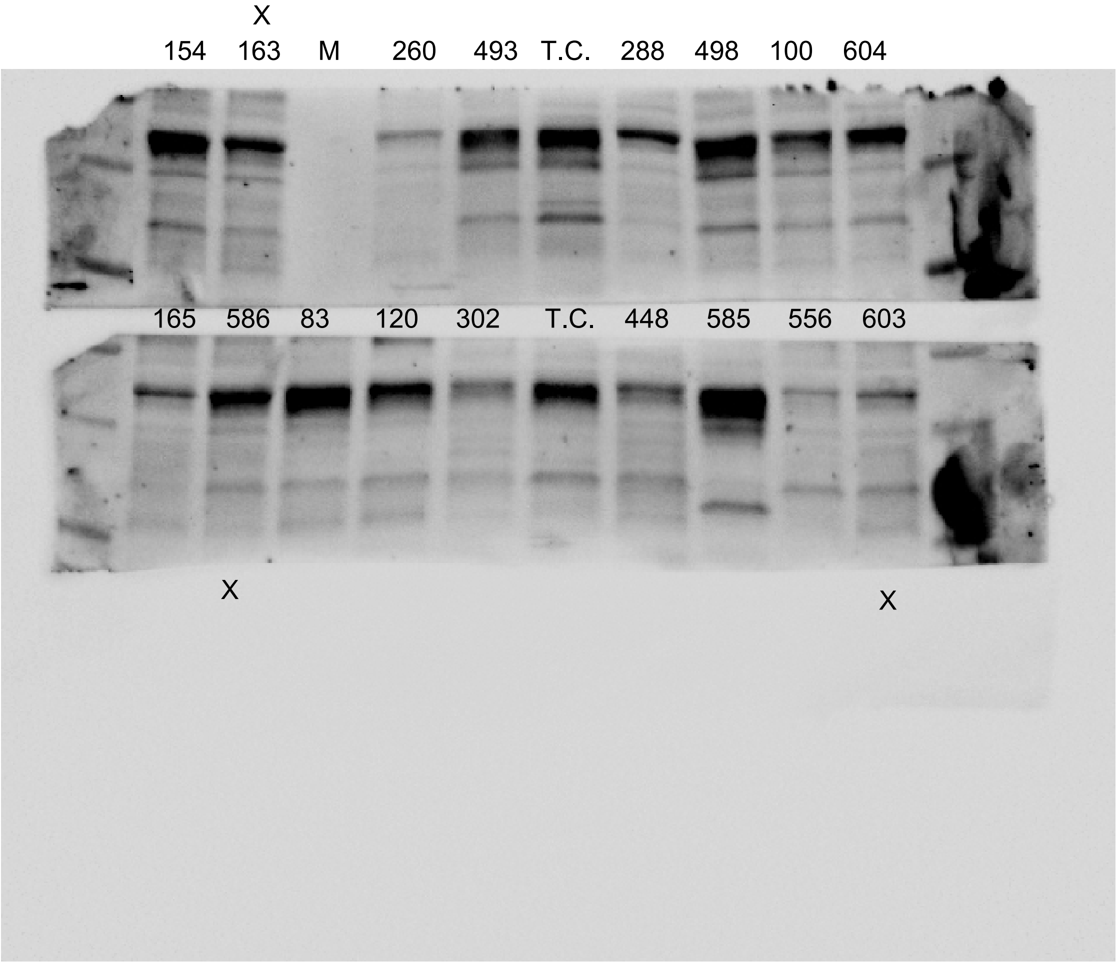

Scan respective to Fig. S1C

Scan respective to Fig. 1C

Capture method: chemiluminescence
Please see page 8, showing additionally the colorimetric signal, from which information on molecular weight ladder was collected
"x" denotes a sample not used in data analysis. However, we have not removed its lane from the Fig1C and FigS1C.
M stands for missing sample; T.C. stands for technical control

Atrogin western blot raw scan

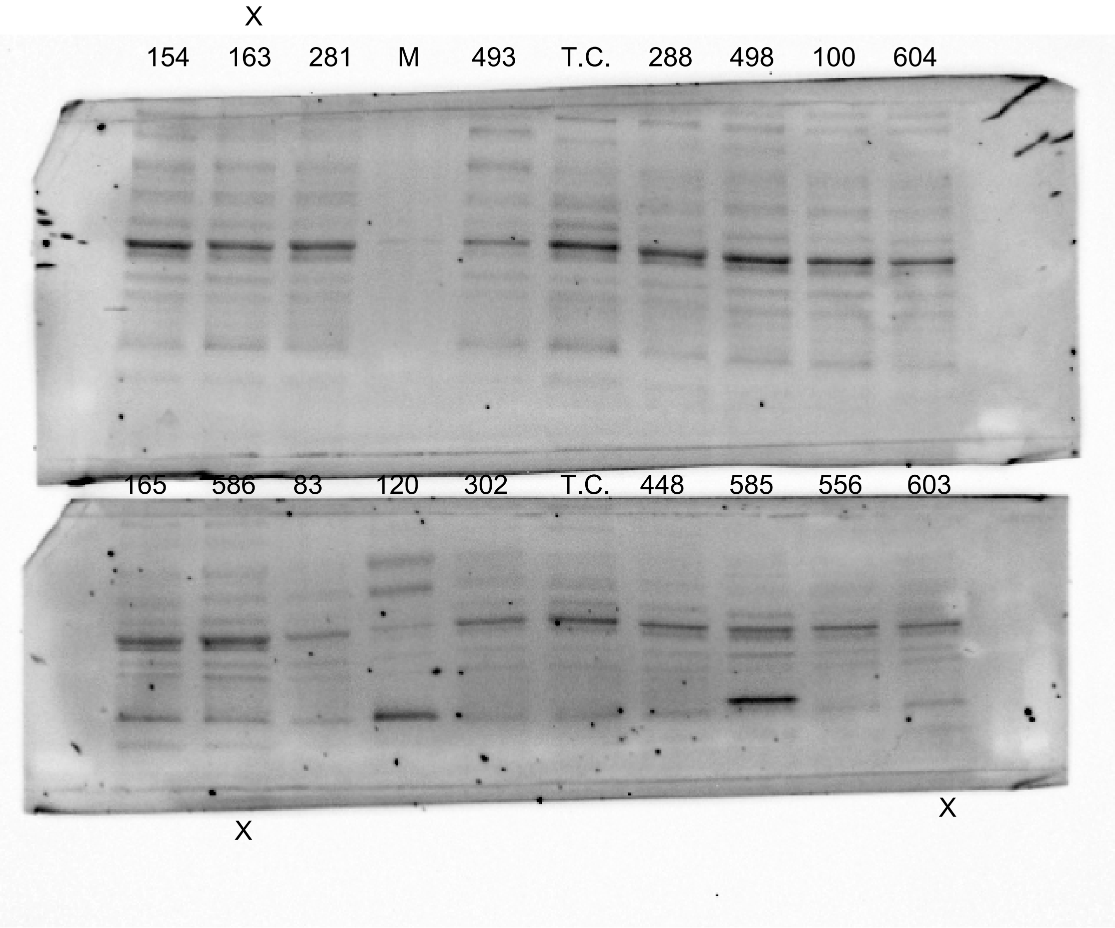

Scan respective to Fig. S1D

Scan respective to Fig. 1D

Capture method: chemiluminescence

Please see page 9, showing additionally the colorimetric signal, from which information on molecular weight ladder was collected

"x" denotes a sample not used in data analysis. However, we have not removed its lane from the Fig1D and FigS1D.

M stands for missing sample, T.C. stands for technical control

MDM2 western blot raw scan

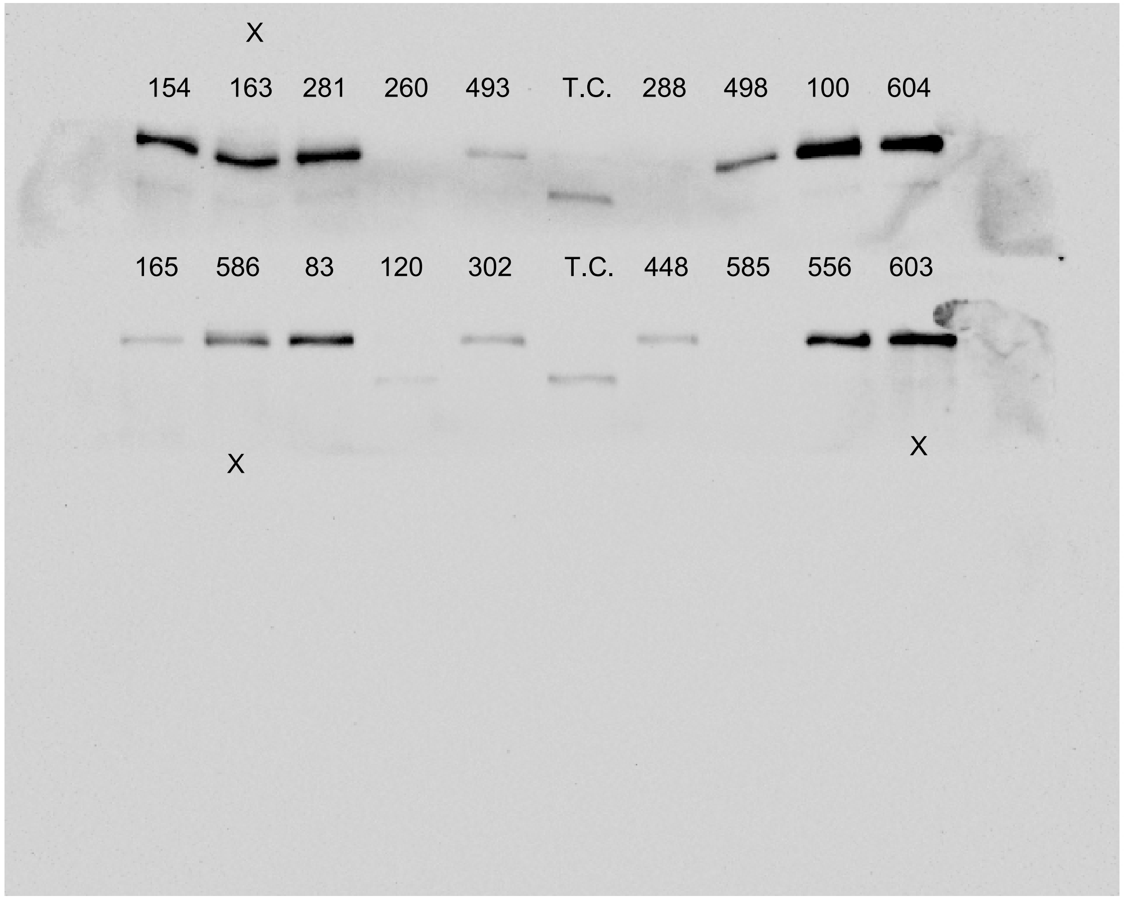

Scan respective to Fig. S1E

Scan respective to Fig. 1E

Capture method: chemiluminescence
Please see page 10, showing additionally the colorimetric signal, from which information on molecular weight ladder was collected
"x" denotes a sample not used in data analysis. However, we have not removed its lane from the Fig1E and FigS1E.
T.C. stands for technical control

Western Blot – Ubiquitin – Raw scans

Images captured with ChemiDoc™ MP

Scan respective to Fig 1A

Scan respective to Fig S1A

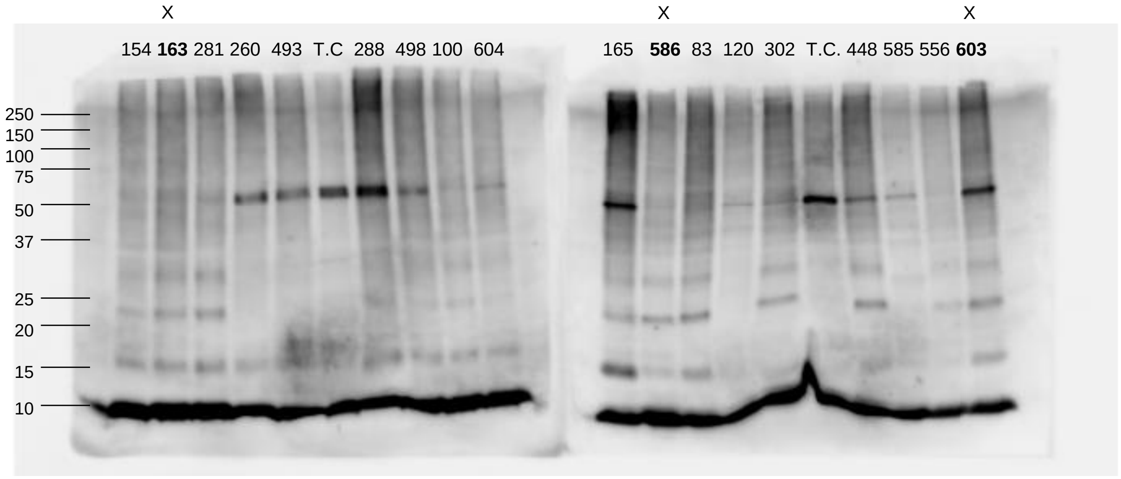

Chemiluminescence signal

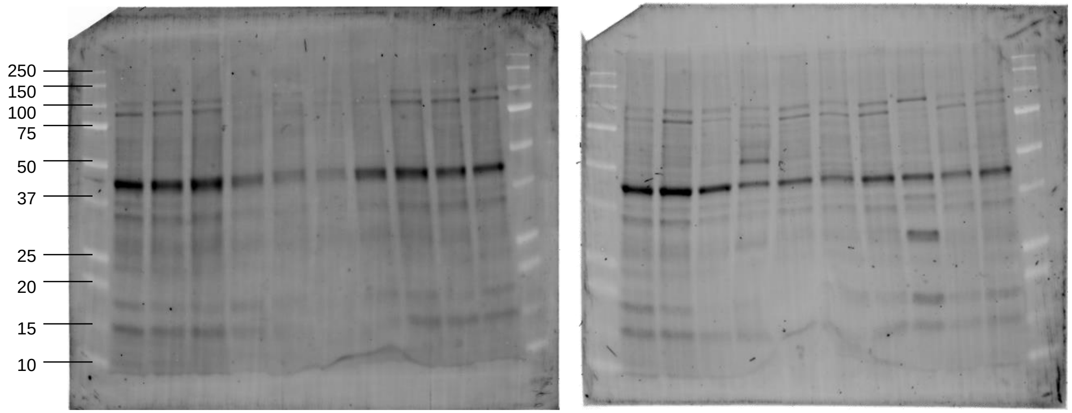

Respective Colorimetric signal

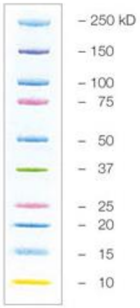

Precision Plus Protein™ Kaleidoscope™ Prestained Protein Standards (Bio-Rad)

In any case, “X” denotes a sample that was dismissed, i.e. densitometric data was not used. The respective lanes have not been cropped out from the Fig 1 and Fig S1. Instead, a justification is given in the caption.

Western Blot – MuRF1 – Raw scans

Images captured with ChemiDoc™ MP

Colorimetric signal of the upper membrane

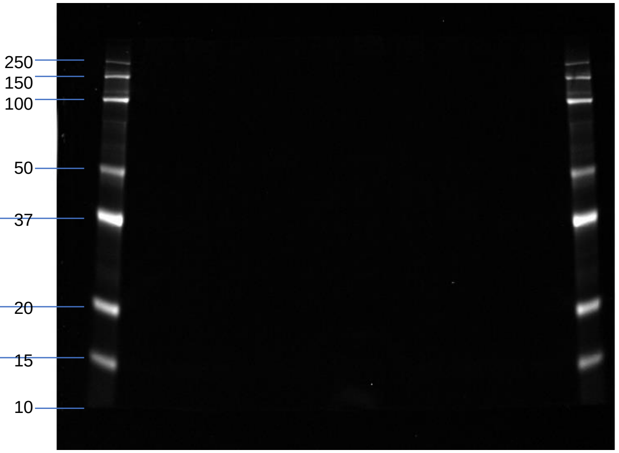

Colorimetric signal of the lower membrane

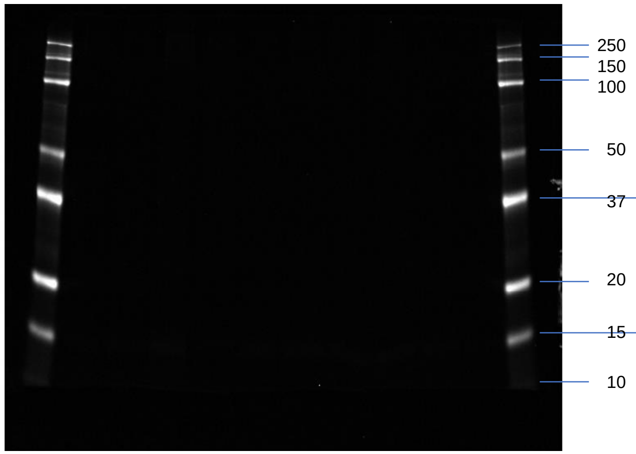

Scan respective to Fig S1B

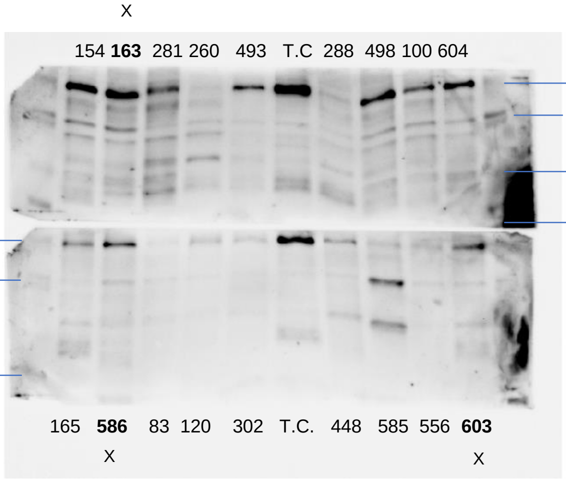

Scan respective to Fig 1B

Chemiluminescence signal (the upper and lower membranes were scanned simultaneously)

The pink bands (75 and 25 kDa) are not observed because a "Far Red Epi Illumination" was used as an excitation source

In any case, "X" denotes a sample that was dismissed, i.e. densitometric data was not used. The respective lanes have not been cropped out from the Fig 1 and Fig S1. Instead, a justification is given in the caption.

Western Blot – MuRF3 – Raw scans

Images captured with ChemiDoc™ MP

Colorimetric signal of the
upper membrane

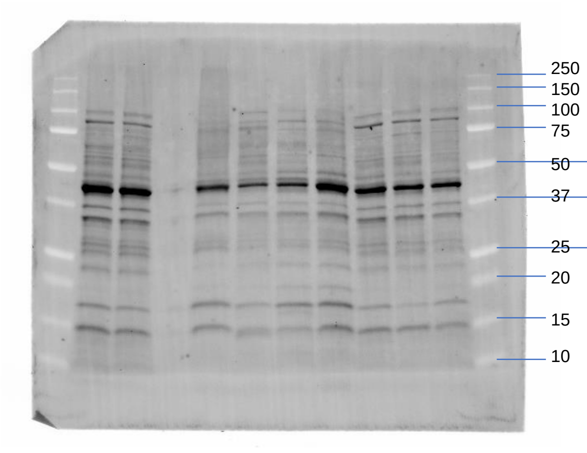

Scan respective to Fig S1C

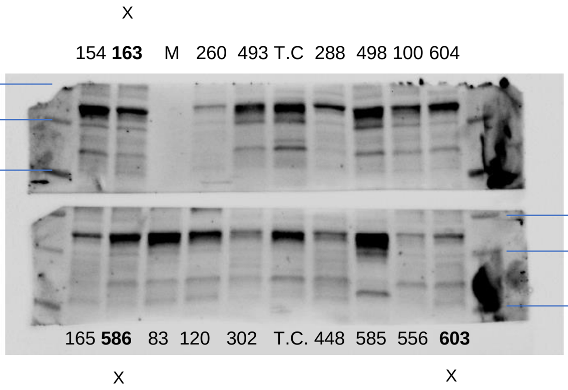

Scan respective to Fig 1C

Chemiluminescence signal
(the upper and lower membranes
were scanned simultaneously)

Colorimetric signal of the
lower membrane

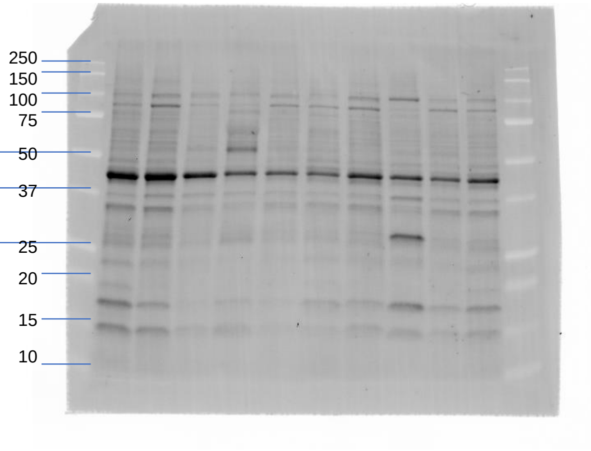

In any case, “X” denotes a sample that was dismissed, i.e. densitometric data was not used. The respective lanes have not been cropped out from the Fig 1 and Fig S1. Instead, a justification is given in the caption.
“M” – missing sample

Western Blot – Atrogin – Raw scans

Images captured with ChemiDoc™ MP

Colorimetric signal of the upper membrane

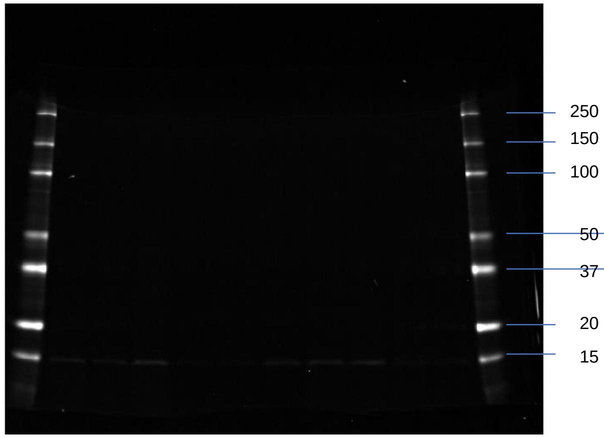

The pink bands (75 and 25 kDa) are not observed because a “Far Red Epi Illumination” was used as an excitation source

Scan respective to Fig S1D

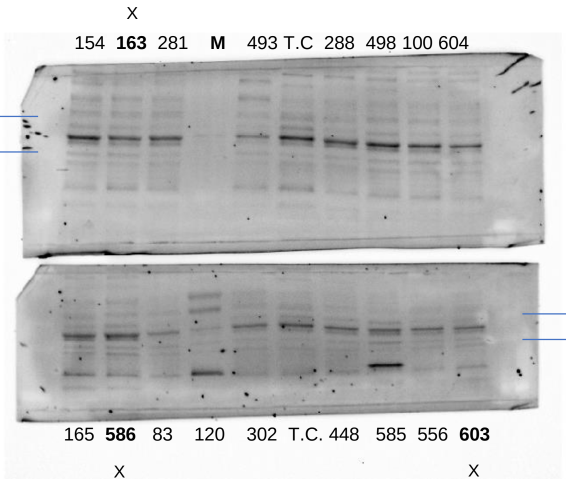

Scan respective to Fig 1D

Chemiluminescence signal
(the upper and lower membranes
were scanned simultaneously)

Colorimetric signal of the lower membrane

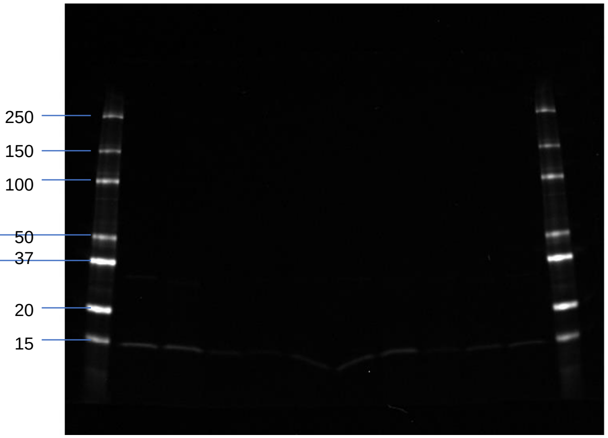

In any case, “X” denotes a sample that was dismissed, i.e. densitometric data was not used. The respective lanes have not been cropped out from the Fig 1 and Fig S1. Instead, a justification is given in the caption.
“M” – missing sample

Western Blot – MDM2 – Raw scans

Images captured with ChemiDoc™ MP

Colorimetric signal of the upper membrane

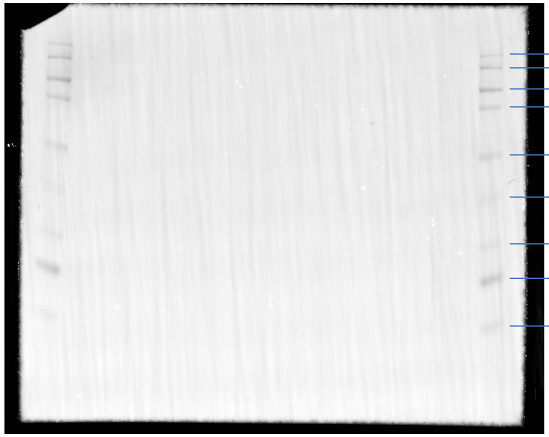

Scan respective to Fig S1E

X

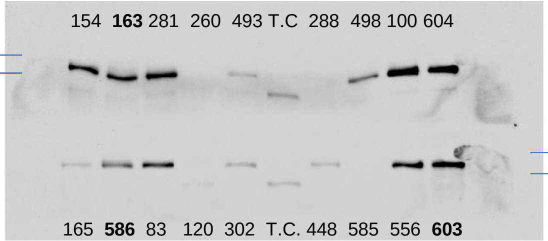

X X

Scan respective to Fig 1E

Chemiluminescence signal
(the upper and lower membranes
were scanned simultaneously)

Colorimetric signal of the lower membrane

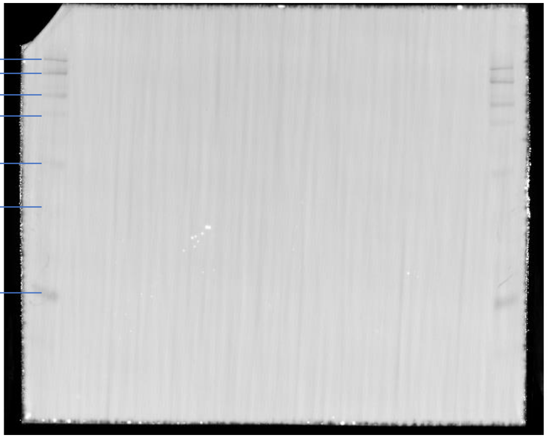

In any case, “X” denotes a sample that was dismissed, i.e. densitometric data was not used. The respective lanes have not been cropped out from the Fig 1 and Fig S1. Instead, a justification is given in the caption.
