# Supplementary material for: Preoperative myocardial expression of E3 ubiquitin ligases in aortic stenosis patients undergoing valve replacement and their association to postoperative hypertrophy
Source: PLoS One. 2020 Sep 18;15(9):e0237000. doi: 10.1371/journal.pone.0237000 (PMC7500680; doi:10.1371/journal.pone.0237000)
Supplement: Attachment — Submitted filename: Response to Reviewers.docx [file pone.0237000.s003.docx]

Response to Reviewers

R.: *We would like to thank the reviewer’s comments. Please find the answers to the points raised in the end of the document.*

Reviewers' comments:

Reviewer's Responses to Questions

Comments to the Author

1. Is the manuscript technically sound, and do the data support the conclusions?

The manuscript must describe a technically sound piece of scientific research with data that supports the conclusions. Experiments must have been conducted rigorously, with appropriate controls, replication, and sample sizes. The conclusions must be drawn appropriately based on the data presented.

Reviewer #1: Yes

2. Has the statistical analysis been performed appropriately and rigorously?

Reviewer #1: Yes

3. Have the authors made all data underlying the findings in their manuscript fully available?

The PLOS Data policy requires authors to make all data underlying the findings described in their manuscript fully available without restriction, with rare exception (please refer to the Data Availability Statement in the manuscript PDF file). The data should be provided as part of the manuscript or its supporting information, or deposited to a public repository. For example, in addition to summary statistics, the data points behind means, medians and variance measures should be available. If there are restrictions on publicly sharing data—e.g. participant privacy or use of data from a third party—those must be specified.

Reviewer #1: Yes

4. Is the manuscript presented in an intelligible fashion and written in standard English?

PLOS ONE does not copyedit accepted manuscripts, so the language in submitted articles must be clear, correct, and unambiguous. Any typographical or grammatical errors should be corrected at revision, so please note any specific errors here.

Reviewer #1: Yes

5. Review Comments to the Author

Please use the space provided to explain your answers to the questions above. You may also include additional comments for the author, including concerns about dual publication, research ethics, or publication ethics. (Please upload your review as an attachment if it exceeds 20,000 characters)

Reviewer #1: The authors set out to identify factors that affect the degree of reverse remodeling following aortic valve replacement. The authors specifically focus their analysis on the ubiquitin-proteasome system (UPS) by measuring preoperative levels of total ubiquitination and several E3 ubiquitin ligases. Pre and postoperative echocardiography data were used to segregate patients into incomplete or complete reverse remodeling groups. While no significant differences in UPS components were observed between these groups, there were significant correlations arising among various cardiac parameters and the UPS. Interestingly, total preoperative ubiquitination levels correlated with increased markers of hypertrophy, while individual E3 ligases had both positive and inverse correlations. Importantly, the authors directly address the limitations of this data set ie. Lack of postoperative protein levels. This data while correlative has the potential, with further study, to identify therapeutic targets and/or predict patient outcomes.

Comments:

1. Most of the data from table 3 is duplicated in figure 2. Should choose one or the other for publication.

R.: *We acknowledge that data is duplicated. To avoid redundancy, we chose to keep Table 3 and dismissed the figure 2 from the manuscript.*

2. Why are some non-significant correlations listed in table 3 and others are left blank?

R.: *We understand the reviewer’s point. We were trying to illustrate that MDM2 was, out of the five molecules quantified, the one with a closer association to left ventricle mass regression. However, we understand that it may be perceived as an incoherent method for data display. Thus, we revised Table 3, and only depicted significant (p < 0.05) and marginally significant correlations (p ≤ 0.10), the latter marked in grey. Thank you for help improving the clarity of the manuscript.*
